# Green Synthesis and Particle Size Control of High-Purity Alumina Based on Hydrolysis of Alkyl Aluminum

**DOI:** 10.3390/ma18092100

**Published:** 2025-05-03

**Authors:** Shuang Zheng, Yao Lu, Huanyu Zhao

**Affiliations:** School of Materials Science and Engineering, Shenyang University of Chemical Technology, Shenyang 110142, China

**Keywords:** aluminum isopropoxide, high purity alumina, hydrolysis, scavenging of impurities

## Abstract

This study introduces a green synthesis strategy for producing high-purity alumina (≥99.99%) through the controlled hydrolysis of aluminum isopropoxide, coupled with a novel impurity removal protocol to address persistent challenges in conventional methods, such as residual silicon/iron impurities and particle agglomeration. The experimental results indicate that La_2_O_3_, 1-(2-pyridylazo)-2-naphthol (PAN), and phenolphthalein exhibit effective removal capabilities for silicon/iron impurities. The addition of 1 wt% La_2_O_3_ reduces silicon content from 99.7 ppm to 16.4 ppm, whereas 0.6 wt% PAN and 0.2 wt% phenolphthalein, employed as iron-binding agents, lower iron content from 66.4 ppm to 20.7 ppm and 9.7 ppm, respectively. Through optimized dropwise hydrolysis and subsequent calcination at 1200 °C for 4 h, nanosized alumina powders with uniform morphology and controlled particle sizes (274–832 nm) were successfully synthesized. The proposed method offers a scalable and efficient pathway for synthesizing high-purity alumina with tailored particle characteristics.

## 1. Introduction

High-purity alumina, defined as ultrafine alumina powder with a purity grade of 4N (mass fraction exceeding 99.99%) [1], has garnered significant attention due to its critical role in advanced industrial applications [2,3]. The proliferation of renewable energy technologies and advancements in electronics and information technology have driven sustained growth in global demand for this material. Due to its exceptional properties, this material plays a critical role in numerous fields, such as LED sapphire substrate [4,5], transparent ceramics [6,7], catalytic carriers [8,9], biomedical implants [10,11], and lithium battery separators [12,13]. The performance of applications in these fields shows a significant positive correlation with alumina purity levels. When metal impurity content is higher, it can lead to defects such as increased dielectric loss, reduced thermal stability, and compromised material performance [14]. In lithium battery separator applications, elevated metal impurity levels may also trigger lithium dendrite formation [15], negatively impacting battery efficiency and safety. Consequently, research into efficient and controllable production processes for high-purity alumina products has become an active area of focus in materials science.

Conventional preparation processes, exemplified by the Bayer method, for example, the extraction of sodium aluminate from bauxite, subsequent crystallization to yield aluminum hydroxide [16], and final calcination to generate alumina, encounter challenges in attaining purity levels of 99.99%. The resultant alumina is constrained by inherent limitations in raw material quality and procedural inefficiencies, relegating its primary applications to refractory materials [17,18], spark plugs [19], and integrated circuit boards. Contemporary methodologies for synthesizing high-purity alumina encompass techniques [20,21], ammonium aluminum sulfate pyrolysis, ammonium aluminum carbonate pyrolysis, hydrothermal synthesis [22,23], activated aluminum hydrolysis [24], and alcohol salt hydrolysis [25]. Among these, the method necessitates specialized high-temperature and high-pressure instrumentation, exhibits prolonged production cycles [26,27], and frequently produces agglomerated crystalline structures. Similarly, ammonium aluminum sulfate and ammonium aluminum carbonate pyrolysis entail intricate operational protocols, bespoke equipment, and extended processing durations necessitating stringent control parameters. Hydrothermal synthesis, while effective, demands sophisticated infrastructure and confronts scalability limitations. Activated aluminum hydrolysis, though technically viable, entails elevated costs and procedural complexity [24]. In contrast, alcohol salt hydrolysis avoids overreliance on mineral resources [28]. This method needs moderate reaction parameters and diminished energy requirements furthermore, it enables the closed-loop recovery of solvent, which aligns with the foundational tenets of contemporary sustainable resource management strategies [29]. The method employs organometallic precursors, e.g., aluminum isopropoxide, and achieves precise control over hydrolysis condensation reactions, theoretically yielding submicron alumina powders with minimal agglomeration [30].

The purity of alumina prepared by the hydrolysis method is closely related to the purity of aluminum salt, and the purity of aluminum salt is affected by the synthesis material. Industrial-grade aluminum raw materials often contain metal impurities such as Fe, Si, V, and B, which may come from the production process of electrolytic aluminum or the recycling process of waste aluminum. For example, iron impurities in aluminum ingots may exist in the form of an alloy. In recent years, research has mainly focused on two key aspects: material purity control and morphology regulation. In terms of purity, raw material purification techniques, such as multi-stage molecular distillation and advanced extraction methods, have been widely adopted to reduce impurity levels in alumina [31,32]. Yin et al. [33] demonstrated a reduction in silicon content within alumina by introducing lanthanum oxide as a desiliconization agent during synthesis. Lanthanides readily form complexes, such as lanthanum isopropoxide, which can further interact to create diol complexes. These complexes distill at 180–220 °C under vacuum (0.1 mmHg) and are easily separated from the original solution via reduced-pressure distillation. Crucially, this scavenging process does not compromise product purity. Grinberg et al. [34] investigated impurity introduction mechanisms during alkoxide synthesis and improved product purity through sequential adsorption and distillation processes. In terms of morphology control, particle morphology and size control can be achieved by adjusting hydrolysis process parameters [35,36]. Wang et al. [37] employed magnesium, aluminum, and n-butanol were employed as raw materials for synthesizing magnesium aluminate alcohol salt utilizing the method. This approach was designed to inhibit the hydrolysis reaction and regulate particle size distribution through precise modulation of hydrolysis kinetics and experimental parameters. Huan [38] achieved γ-alumina with tunable morphologies and pore structures by varying aluminum salt types and water–aluminum molar ratios during alkoxide hydrolysis.

At present, there are several trace impurities in the obtained alumina, among which the silicon and iron content are relatively high and difficult to remove. Most people choose to add a scavenging agent to reduce the content of silicon or iron, and the purification effect of alumina is limited. Hydrolysis conditions can affect the state of alumina precursors, indirectly leading to the morphology and particle size of alumina particles, resulting in uneven distribution, pore size, and even agglomeration. Building upon this foundation, the present study introduces a systematic “precursor purification–hydrolysis regulation–morphology optimization” framework. This study systematically investigates the selective chelation mechanisms of metal impurities (silicon and iron) using tailored chelating agents to enable efficient impurity removal, while the hydrolysis parameters are adjusted to prepare alumina powder with different particle sizes to meet the performance requirements of different application fields. For example, in a lithium battery diaphragm, different ceramic particle sizes also have different effects on the electrolyte infiltration, ionic conductivity, and battery cycle performance of the diaphragm [39].

## 2. Materials and Methods

### 2.1. Raw Materials

In this study, aluminum particles (purity: 99.5; average diameter: 1 mm) were sourced from Hebei Lebo Materials Co., Ltd. (Hebei, China). Analytical-grade reagents, including C_3_H_7_OH (≥99.9%), AlCl_3_ (≥99.9%), and La_2_O_3_ (≥99.9%), were procured from Shandong Keyuan Biochemical Co., Ltd. (Shandong, China). Additional reagents, such as ethylene diamine tetraacetic acid (EDTA, ≥99.5%), cyclohexane diamine tetraacetic acid (CDTA, ≥99.9%), 1-(2-pyridylazo)-2-naphthol (PAN, ≥99.9%), and phenolphthalein (PH, ≥99.9%), were supplied by Shanghai Aladdin Biochemical Technology Co., Ltd. (Shanghai, China).

### 2.2. Experimental Method

In this experiment, commercially available aluminum pellets were used as the primary reactant, isopropanol served as the reagent for synthesizing aluminum isopropoxide, and anhydrous aluminum chloride was selected as the catalyst. Deionized water was employed in the hydrolysis process. Given the hygroscopic nature of both isopropanol and anhydrous aluminum chloride, the synthesis reaction was conducted in a dry environment. As the reaction between aluminum chloride and isopropanol is exothermic and generates hydrogen gas, the pressure and liquid phase of the reaction environment may fluctuate. To minimize adverse effects on the synthesis, the aluminum chloride catalyst was added to isopropanol at room temperature and stirred until complete dissolution. Silica-scavenging agents La_2_O_3_ and iron-scavenging agents, e.g., PAN, EDTA, CDTA, and PH, were introduced during synthesis to reduce impurities. La_2_O_3_ can be used to form a high-boiling-point substance with silicon, thereby removing Si from the system. Scavenging agents can form stable complexes with iron ions under specific conditions, thereby removing or stabilizing them from the environment. Their molecular structures contain specific functional groups such as carboxyl, hydroxyl, and amino groups. These groups form coordination bonds with iron ions, generating stable cyclic chelates [40]. Due to the exothermic nature of the reaction and hydrogen production, reflux condensers were attached to the two ends of the three-necked flask to prevent flash boiling. The vessel was immersed in an oil bath at 80 °C until the aluminum particles were completely consumed, yielding a black, mixed-phase liquid. The mixture underwent distillation to evaporate and recover excess isopropanol, followed by vacuum distillation to collect the 130–140 °C fraction at 0.01 MPa, producing transparent and colorless aluminum isopropoxide colloids. Subsequently, hydrolysis was achieved via the dropwise addition of an aqueous solution, and the product was oven-dried at 100 °C. Finally, the dried powder was placed in a crucible, transferred to a muffle furnace, and calcined at 1200 °C for 4 h to obtain high-purity aluminum oxide (Al_2_O_3_) powder.

### 2.3. Testing Methods

This study employed a Fourier Transform Infrared (FTIR) spectrometer (model Nicolet IS 10, Thermo Fisher Scientific) to characterize the functional groups of aluminum isopropanolate. A liquid sample was applied to a potassium bromide (KBr) slide. The spectral range was set from 400 to 4000 cm^−1^, with a resolution of 0.4 cm^−1^, and 32 scans were performed. Elemental analysis was performed using an Agilent 7500 CE ICP-MS (Agilent Technologies, Santa Clara, CA, USA). The instrument was operated under the following conditions: peristaltic pump speed was set at 50 r/min, nebulizer gas flow rate was 0.5 L/min, argon plasma gas flow rate was 15.00 L/min, and the radio frequency power was maintained at 1150 W. Phase identifications were performed by X-ray diffraction (XRD, Tongda TD-3500 instruments, Dandong, China) using nickel-filtered Cu Kα radiation (30 kV, 20 mA) over a 2θ range of 10°–80° with a scanning speed of 4°/min. The nanoparticle size analyzer (NanoSizer; SZ902, Linke Optical Scientific Instruments Co., Ltd., Fuzhou, China) was used to determine the particle size distribution of the powder. The morphologies were investigated via scanning electron microscopy (SEM; Regulus 8100, Hitachi, Tokyo, Japan) under a voltage of 15 kV.

## 3. Results and Discussion

### 3.1. Synthesis of Aluminum Isopropoxide


(1) $$2Al+6C_{3}H_{7}OH=2Al{(C}_{3}H_{7}{O)}_{3}+3H_{2}↑$$

(2) $$2Al(C_{3}H_{7}{O)}_{3}+xH_{2}O={Al}_{2}O_{3}\cdot xH_{2}O+6C_{3}H_{7}OH$$

(3) $${Al}_{2}O_{3}{\cdot xH}_{2}O≜{Al}_{2}O_{3}+xH_{2}O$$

Aluminum and isopropanol react in the presence of a catalyst at room temperature and pressure to form aluminum isopropoxide and hydrogen gas, releasing heat. The reaction does not produce toxic or hazardous substances aside from hydrogen. During hydrolysis, aluminum isopropoxide reacts with hydroxyl groups from water molecules to form hydrated aluminum oxide. The hydrolyzed product is dried and calcined to remove water from crystallization, yielding high-purity alumina. Unreacted isopropanol and the by-product isopropanol generated during hydrolysis can be recovered and reused via distillation, reducing raw material consumption, waste generation, and production costs. The entire production process avoids strong acids, bases, or toxic reagents. The only by-products are isopropanol and water, preventing the release of sulfur dioxide, nitrogen oxides, or heavy metal pollutants. This method is environmentally friendly and aligns with sustainable development principles.

#### 3.1.1. FTIR Analysis

**Figure 1:**
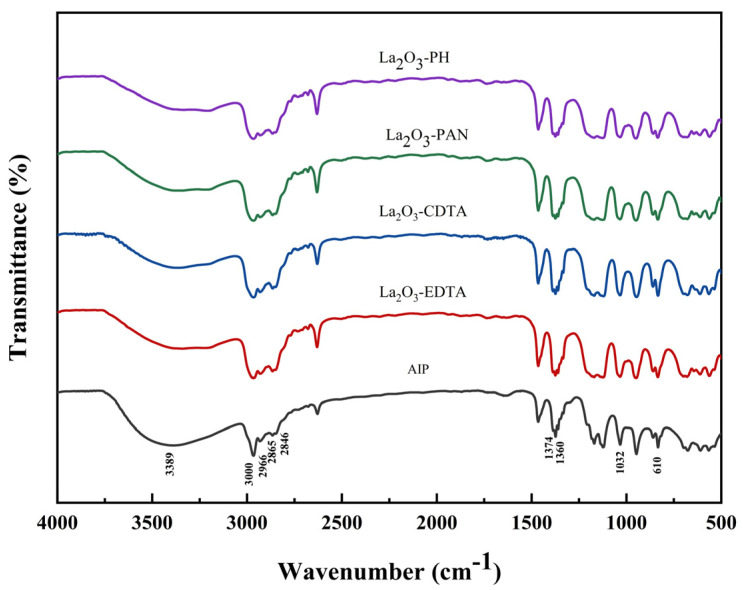
FTIR analysis of purified aluminum isopropoxide with different scavenging agents.

Figure 1 presents the infrared spectra of the synthesized products. These products include aluminum isopropoxide and purified aluminum isopropoxide with various additives, namely La_2_O_3_ combined with EDTA, CDTA, PAN, and PH. The broad absorption peak at 3388.9 cm^−1^ corresponds to the O-H stretching vibration band, which is caused by the hydrolysis of aluminum isopropanol on the surface of the product during KBr tablet pressing due to contact with water in the air. Peaks near 3000, 2966, 2865, 2846, 1374, and 1360 cm^−1^ represent characteristic isopropyl skeletal vibrations. The peak at 1032cm^−1^ indicates C–O stretching vibrations linked to aluminum, while the peak at 610 cm^−1^ corresponds to asymmetric Al–O bond stretching vibrations. These results confirm the presence of Al–O groups, isopropyl groups, and Al–O–C linkages in the synthesized compound. Notably, the infrared spectra of the samples with scavenging agents exhibit no new functional groups, and all characteristic peaks align with those of the scavenger-free sample. This indicates that the additives did not alter the chemical composition or properties of the synthesized aluminum isopropoxide.

#### 3.1.2. Elemental Content Analysis

**Figure 2:**
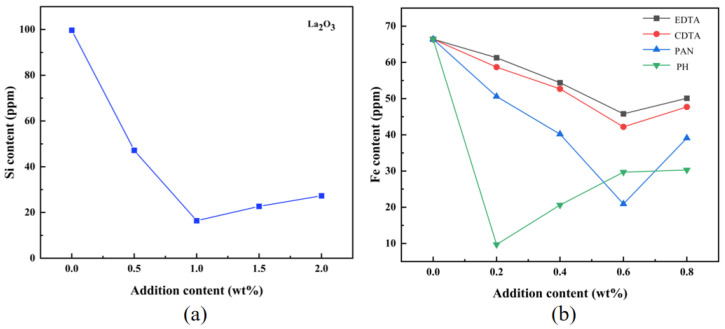
The influence of additive content on the elemental content in the product: (**a**) silicon content after adding La_2_O_3_; (**b**) iron content in aluminum isopropoxide after adding EDTA, CDTA, PAN, and PH.

Aluminum isopropoxide is a critical precursor for synthesizing high-purity alumina, as its impurity content directly impacts the purity of alumina. Purity analysis of the synthesized aluminum isopropoxide was conducted without any scavenging agents, and it contained high levels of iron and silicon impurities. Reducing silicon and iron impurities is closely related to the improvement in alumina product purity. Therefore, the addition of impurity-scavenging agents during aluminum isopropoxide synthesis was investigated. The effects of a silica scavenger (La_2_O_3_) and an iron scavenger on reducing silicon and iron content in the product were analyzed. These results were compared with scavenger-free aluminum isopropoxide to determine the optimal additive ratios for decontamination (Figure 2).

The silicon content in the product decreased significantly after adding La_2_O_3_, dropping from 99.7 ppm to 16.4 ppm at a mass ratio of 1%. This demonstrates that La_2_O_3_ effectively reduces silicon impurities during synthesis. Based on the coordination chemistry characteristics of lanthanide metals, their silicon removal mechanism can be attributed to the tendency for multi-dentate coordination. Taking lanthanum isopropoxide as an example, the central La^3^+ has an empty f-orbital with high charge density, making it easy to bond with silicon tetrahedra, thus achieving the directional removal of silicon elements. Additionally, lanthanum isopropoxide also has strong coordination ability, allowing it to form secondary coordination with other metals. After adding EDTA and CDTA, at a mass ratio of 0.6%, the iron content was minimized; however, while CDTA demonstrated better iron scavenging efficacy than EDTA, both additives underperformed in the experimental conditions. EDTA and CDTA contain amine nitrogen and carboxyl oxygen functional groups, which grant these reagents strong coordination capabilities. However, in the alkaline isopropanol environment, multiple competing ions (e.g., Cu^2+^, Co^2+^, and Ni^2+^) were present alongside iron ions before complexation. In addition, Al^3+^, Fe^2+^, and Fe^3+^ ions contributed to significant complexation competition. Based on the stability constants of the resulting metal complexes, CDTA forms more stable complexes than EDTA.

**Figure 3:**
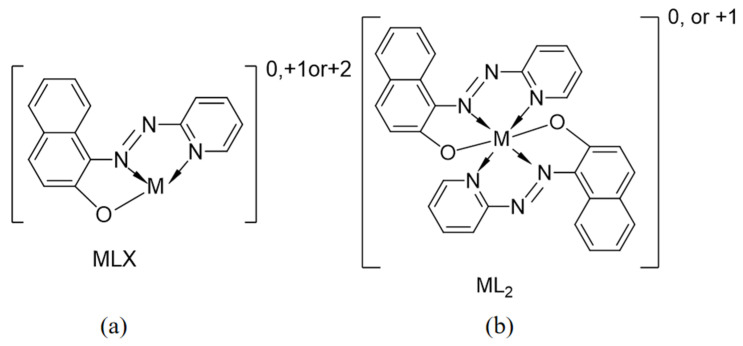
The coordination geometry of PAN complexes with metal ions: (**a**) MLX-type; (**b**) ML2-type.

With the addition of PAN, the iron content in aluminum isopropoxide initially decreases, then increases, reaching its minimum at a mass ratio of 0.6%. The molecular structure of PAN contains nitrogen and oxygen coordinating atoms. The azo group in PAN participates in chelation, forming a ring structure that establishes conjugation with the benzene ring, thereby enhancing the stability of the chelates. PAN forms MLX-type chelates with metal ions that have a coordination number of 4 and conventional ML_2_-type chelates with those that have a coordination number of 6 (Figure 3). When PAN is added in small amounts, some Fe^2+^ or Fe^3+^ ions remain unchelated. Conversely, excess PAN provides an overabundance of functional atoms, enabling PAN to act as a bidentate ligand for iron ions. However, steric hindrance destabilizes the bidentate chelates formed between PAN and iron ions. Under reduced-pressure distillation conditions, steric hindrance disrupts the chelates’ chemical equilibrium, resulting in a gradual increase in the product’s iron content [40].

**Figure 4:**
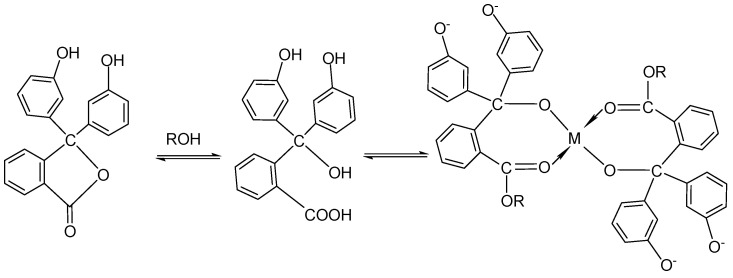
Presumed structure of phenolphthalein chelate with iron ions.

When PH is added at a mass ratio of 0.2%, the product exhibits the lowest iron content, indicating that phenolphthalein is one of the most effective iron removal agents. In the isopropanol–aluminum isopropoxide system, the PH of the alcohol solution facilitates alcoholysis, opening the lactone structure of phenolphthalein and forming a quinoidal structure. During this process, the phenolic hydroxyl and carboxylic acid groups within the phenolphthalein molecule can coordinate with Fe^3+^ ions, forming insoluble and stable chelates that effectively remove iron ions from the system. However, the excess coordination groups provided by phenolphthalein can form stable, soluble complexes with Fe^3+^. This leads to an increase in the concentration of iron ions in the system. This structural change is accompanied by a color transition from colorless to red. The color shift visually indicates the occurrence of the lactone ring-opening reaction. The open intermediate structure contains hydroxyl and carboxyl oxygen atoms, which chelate iron ions to form highly stable complexes [41] (Figure 4).

To improve the purity of the alumina products, both iron and silicon impurities are simultaneously removed by adding a silica-scavenging agent and an iron-scavenging agent during the synthesis of aluminum isopropoxide. This purification process achieves alumina purity levels exceeding 99.99%. As shown in Table 1, the ideal combinations involve La_2_O_3_ with PAN and La_2_O_3_ with PH. Notably, when PH at a mass ratio of 0.2% and La_2_O_3_ at a mass ratio of 1% are co-added, the synthesized product reaches a purity of 99.99337%, marking the highest purity observed in this study.

### 3.2. Hydrolysis of Aluminum Isopropoxide

**Figure 5:**
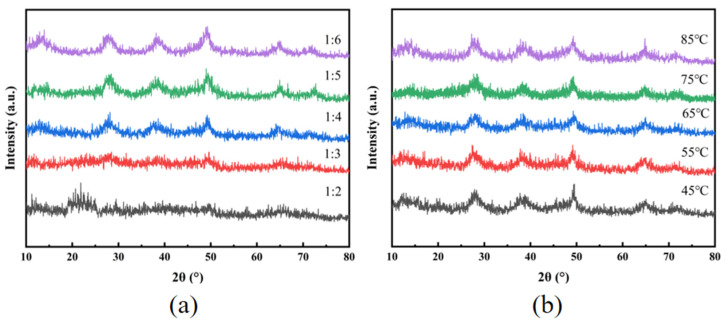
XRD spectra of hydrolysis products: (**a**) different water consumption; (**b**) different temperatures.

Figure 5a presents the X-ray diffraction (XRD) spectra of the aluminum isopropoxide hydrolysis products under varying water–aluminum isopropoxide molar ratios ranging from 1:2 to 1:6. During hydrolysis, the crystallinity of the products progressively increased with higher water content. At molar ratios of 1:2 and 1:3, the XRD spectra showed broad, low-intensity peaks, signifying incomplete hydrolysis caused by insufficient water. However, at higher molar ratios (1:4, 1:5, and 1:6), the spectra exhibited sharp, narrow peaks with uniform intensity, indicating complete hydrolysis and consistent crystallinity regardless of additional water. This demonstrates that excess water facilitates full hydrolysis without modifying the crystalline structure of the product.

Figure 5b depicts the X-ray diffraction (XRD) spectra of the hydrolysis products obtained at different hydrolysis temperatures. Temperature influences the free energy of solutes, nucleation rate, and growth rate of the products. It also governs the reaction kinetics, condensation of intermediates, and crystallization dynamics, ultimately determining the crystal morphology, crystallinity, and microstructure of the final material. Experimental studies using aluminum isopropoxide hydrolyzed with excess water at five temperatures (45–85 °C; 10 °C intervals) revealed consistent phase composition across all the conditions. Despite varying temperatures, aluminum isopropoxide reacts with hydroxyl groups from water molecules to form hydrated aluminum oxide, demonstrating that temperature during hydrolysis had no significant impact on the crystal phase.

### 3.3. Calcination of Hydrolysis Products

#### 3.3.1. XRD Analysis

**Figure 6:**
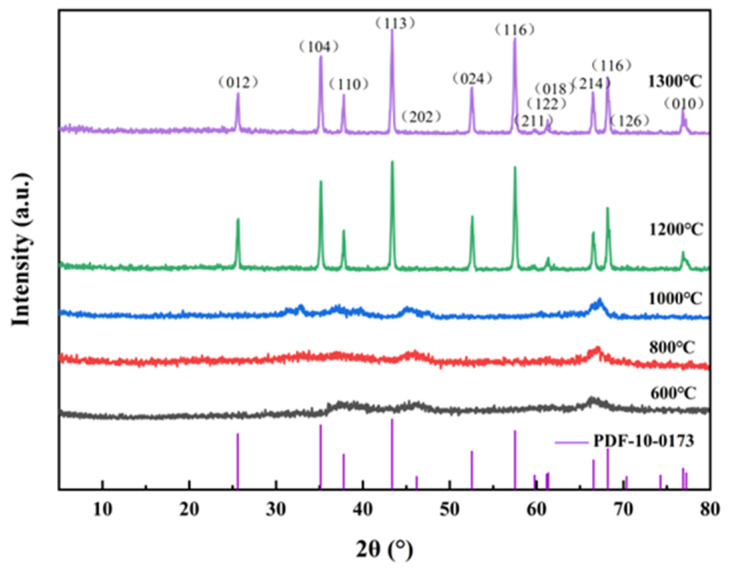
XRD spectra of high-purity alumina at different calcination temperatures.

Figure 6 depicts the X-ray diffraction (XRD) spectra of the calcined products synthesized at different temperatures and held for 4 h. The XRD peak profiles shifted markedly with increasing calcination temperature, reflecting significant changes in crystallinity. At temperatures below 1000 °C, the peaks appeared broad and low-intensity, indicating poorly crystalline phases and incomplete phase stabilization. Higher temperatures promoted the formation of thermodynamically stable phases, as evidenced by sharper peaks. Above 1200 °C, the peaks became narrow and intense, closely matching the reference pattern for α-Al_2_O_3_ (PDF#10-0173). However, the XRD peaks at 1200 °C and 1300 °C were indistinguishable, confirming that complete transformation to pure α-alumina occurs when the material is held at 1200 °C for 4 h.

#### 3.3.2. Particle Size and Morphology Analysis

**Figure 7:**
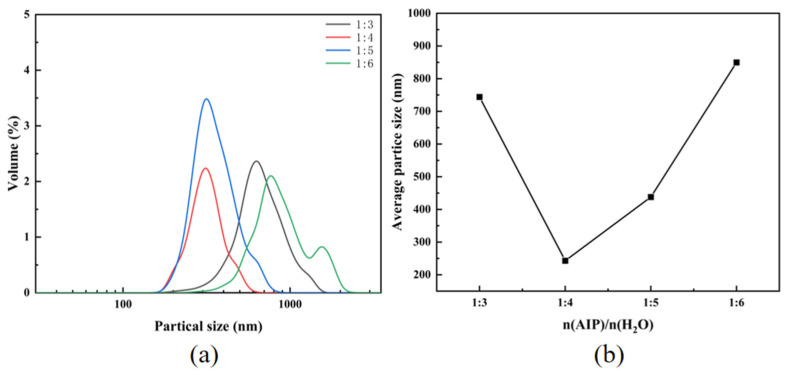
(**a**) Particle size distribution of alumina; (**b**) average particle size profile of alumina.

Figure 7 illustrates the particle size distribution and average particle size curve of the alumina synthesized under varying water–aluminum isopropoxide ratios. Aluminum isopropoxide was hydrolyzed under reflux conditions at 85 °C with the dropwise addition of water, followed by calcination at 1200 °C for 4 h to yield high-purity alumina powder. The data reveal that increasing water content broadens the particle size distribution of alumina, while the average particle size curve exhibits an initial decrease followed by an increase. The particle size and morphology of alumina are strongly influenced by the structural properties of hydrolysis intermediates. At low water conditions with *n*(AIP):*n*(H_2_O) = 1:3, hydrolysis proceeds sluggishly due to limited hydroxyl group availability. This slow reaction promotes the partial polymerization of aluminum isopropoxide, resulting in low cross-linking density and irregular particle growth. Consequently, the average particle size is inconsistently modulated. In contrast, sufficient water, such as *n*(AIP):*n*(H_2_O) = 1:4 or 1:5, accelerates hydrolysis, enabling rapid nucleation and high nucleation density. The rapid solute depletion shortens crystal growth time, yielding smaller, more uniform particles. Excess water further reduces system viscosity, enhances mass transfer efficiency, and minimizes local concentration gradients, fostering homogeneous nucleation and narrow particle size distributions. However, under excessive water conditions such as *n*(AIP):*n*(H_2_O) = 1:6, residual water persists post-drying, adhering to crystal surfaces via hydrogen bonding with hydroxyl groups. This promotes crystal agglomeration, broadening the particle size distribution, and increasing average particle size.

**Figure 8:**
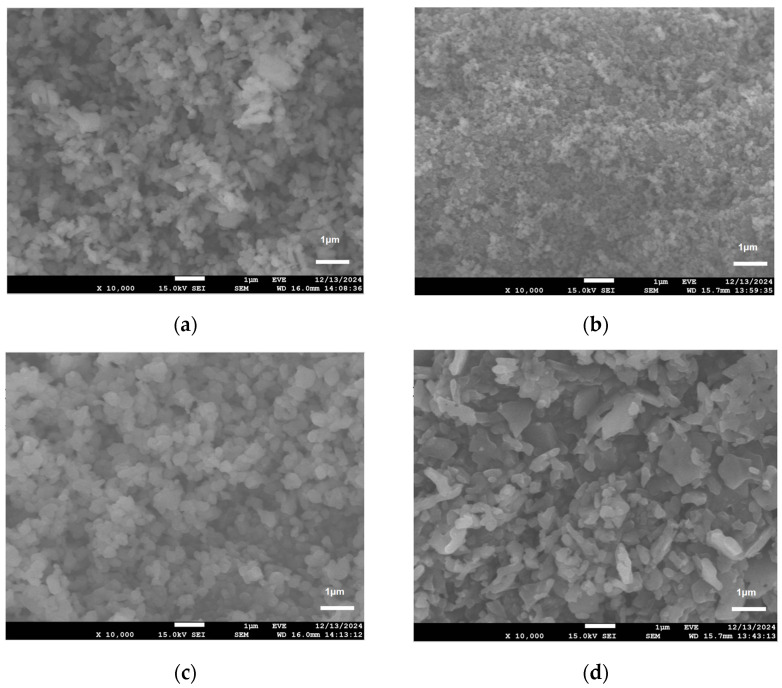
Morphology of alumina prepared with different hydrolysis ratios: (**a**) 1:3; (**b**) 1:4; (**c**) 1:5; (**d**) 1:6.

As shown in Figure 8, the morphology of aluminum oxide varies significantly with particle size. The smallest average particle size (247 nm) exhibits relatively uniform morphology, achieved by the dropwise addition of the hydrolysis solution followed by calcination. Deviations from the optimal water–aluminum isopropoxide ratio significantly affect the product’s particle size. The SEM images reveal that as the molar ratio of aluminum isopropoxide to water increases, the particle size initially decreases and then increases. Within the molar ratio range of 1:3 to 1:5, the particles transition from a partially aggregated state to a more dispersed and uniform distribution, exhibiting relatively uniform shapes. However, when the amount of water is further increased beyond this range, the particle size becomes larger, the shapes become irregular, and significant aggregation occurs. At low water levels, the limited availability of hydroxyl groups restricts hydrolysis, thereby promoting the partial polymerization of aluminum isopropoxide. This leads to a lower crosslink density, irregular particle growth, and aggregation. When the water–aluminum isopropoxide ratio is increased to 1:4 or 1:5, the higher concentration of hydroxyl groups accelerates hydrolysis, increasing nucleation rates and rapidly depleting reactants. This reduces the time available for crystal growth, allowing nucleation to outpace growth. Additionally, the increased water content lowers the system viscosity, enhancing solute mass transfer and favoring the formation of uniform particles. However, excessive water can result in residual moisture post-drying. This residual moisture adheres to the crystals via hydroxyl hydrogen bonding, causing agglomeration, larger particle sizes, and irregular shapes. In summary, low water content results in a slow hydrolysis rate, which promotes the formation of monodisperse nuclei and favors smaller particle sizes. Conversely, while high water content rapidly produces numerous initial nuclei, it simultaneously enhances agglomeration, yielding submicron to micron-sized particles with irregular morphology.

## 4. Conclusions

Within the reaction system, the concurrent addition of iron and silicon scavengers significantly enhances product purity optimization. When 1 wt% La_2_O_3_ is combined with either 0.2% P.H or 0.6% PAN, the synthesized aluminum isopropoxide attains exceptional purity levels exceeding 99.99%. Notably, the PH iron scavenger exhibited superior performance, achieving a remarkable reduction of iron impurities from 66.4 ppm to 9.7 ppm.

Alumina particle size and morphology are precisely controlled by adjusting hydrolysis ratios. Hydrolysis is performed at 85 °C via the drip addition of the hydrolysis solution, followed by drying and calcination. At a 1:4 molar hydrolysis ratio, the resulting alumina exhibits uniform morphology and a narrow particle size distribution, with an average particle size of 247 nm. When the hydrolysis molar ratio is 1:5, the average particle size of the obtained alumina is 432 nm.

## Figures and Tables

**Table 1:** Purity of the synthesized products after the addition of scavenging agents.

Impurity Content (ppm)	Fe	Si	Na	Ca	Mg	Cu	Al (%)
scavengers-free	66.4	99.7	12.9	18.4	8.4	<1	99.97932
CDTA+ La_2_O_3_	45.7	16.5	12.9	18.4	8.4	<1	99.98971
PAN+ La_2_O_3_	20.7	16.4	13.0	18.2	8.3	<1	99.99227
PH+ La_2_O_3_	9.7	16.7	12.8	17.9	8.2	<1	99.99337

## Data Availability

The original contributions presented in this study are included in the article. Further inquiries can be directed to the corresponding authors.
